# A phenomenological exploration of work-related post-traumatic growth among high-functioning adults maltreated as children

**DOI:** 10.3389/fpsyg.2022.1048295

**Published:** 2022-12-22

**Authors:** Avital Kaye-Tzadok, Tamar Icekson

**Affiliations:** ^1^Social Work Department and the Lior Tsfaty Center for Suicide and Mental Pain Studies, Ruppin Academic Center, Emek Hefer, Israel; ^2^Program in Organizational Development & Consulting, School of Behavioral Sciences, Peres Academic Center, Rehovot, Israel; ^3^Departments of Management and Education, Ben-Gurion University of the Negev, Beer-Sheva, Israel

**Keywords:** post-traumatic growth, childhood maltreatment, meaning-making, work, adult survivors

## Abstract

**Introduction:**

Childhood maltreatment is a highly prevalent traumatic experience, and its adverse psychological and behavioral consequences are well-documented. Notwithstanding these adverse outcomes, many individuals who suffered from traumatic experiences report post-traumatic growth, i.e., transformative positive changes resulting from their struggle to cope. Post-traumatic growth has been extensively explored among adult survivors of childhood maltreatment, with findings indicating both the previously recognized domains (personal strength, relating to others, appreciation of life, openness to new possibilities, and spiritual change) as well as abuse-specific domains of growth (e.g., increased ability to protect themselves from abuse). However, little attention has been given to vocational aspects of post-traumatic growth among survivors, despite the central role and importance of work in adulthood. Exploration of post-traumatic growth at work has focused on certain vocational traumatic experiences, such as those which occur in the military, or through secondary trauma. This exploratory qualitative study focuses on the question: What is the lived experience of work-related post-traumatic growth among high-functioning adult survivors of CM?

**Method:**

Twenty in-depth interviews were held with high-functioning working adults who were maltreated as children. Phenomenological analysis was applied to the retrospective data reported in these interviews.

**Result:**

Rich descriptions of work-related positive psychological changes were provided by all participants. Analysis revealed that survivors’ post-traumatic growth corresponded with all five previously recognized domains of growth: changes in self, relating to others, openness to new possibilities, finding meaning to the abuse, and appreciation of life. It also revealed that work is perceived as a form of resistance (a subtheme of changes in self), and that finding meaning entails three emerging subthemes: being a survivor and a role model, giving others what was needed and never received, and making a better world.

**Discussion:**

While the vocational lives of survivors of childhood maltreatment have rarely been examined through the lens of post-traumatic growth, our results show this lens to be highly valuable. Work-related post-traumatic growth has relevance not only regarding vocational traumas occurring in adulthood as has been previously studied, but also in the context of childhood traumas. Moreover, our research broadens the understanding of the possible domains of work-related growth.

## Introduction

Childhood maltreatment is a highly prevalent traumatic experience, and its adverse psychological and behavioral consequences are well-documented (Fergusson et al., 2013; Carr et al., 2013; Gardner et al., 2019). Notwithstanding these adverse outcomes, a recent review has shown that nearly 50% of individuals who suffered from traumatic experiences report moderate to high levels of post-traumatic growth (PTG), i.e., transformative positive changes resulting from the struggle to heal from trauma (Wu et al., 2019). Such changes are mostly categorized by the following domains: personal strength, relating to others, appreciation of life, openness to new possibilities, and spiritual change (Calhoun and Tedeschi, 2001).

PTG has been extensively explored among adult survivors of childhood maltreatment, with reference to both the previously recognized domains (personal strength, relating to others, appreciation of life, openness to new possibilities, and spiritual change) as well as abuse-specific domains of growth, such as an increased ability to protect themselves from abuse (McMillen et al., 1995; Kaye-Tzadok et al., 2016; Schaefer et al., 2018; Lahav et al., 2020; Tranter et al., 2021). However, little attention has been given to work-related aspects of PTG among survivors, despite the central role of work and its importance in adulthood. Prior exploration of PTG at work has focused mainly on two major types of traumatic experiences (Maitlis, 2020): first, traumatic experiences occurring at work, such as in military service or among firefighters and second, PTG resulting from coping with secondary trauma, such as among helping professionals. The current study offers an opportunity to extend and complement the literature regarding work-related PTG. Building on the growth domains originally suggested by Tedeschi and Calhoun (1996), this exploratory qualitative study focuses on the question: What is the lived experience of work-related post-traumatic growth among high-functioning adult survivors of CM?

## Literature review

### Childhood maltreatment

Child maltreatment (CM) is an alarmingly prevalent public health issue and social problem. Using data derived from self-report studies carried out in several countries across the globe, estimated prevalence rates are 36% emotional abuse, 23% physical abuse, 16%–18% neglect, and 13% sexual abuse, suggesting CM is widespread (e.g., Stoltenborgh et al., 2015). CM is defined by the World Health Organization (2020) as:

Abuse and neglect that occurs to children under 18 years of age, which includes all types of physical and/or emotional ill treatment, sexual abuse, neglect … and which results in actual or potential harm to the child’s health, survival, development or dignity in the context of a relationship of responsibility, trust or power.

The deleterious effects of CM on children’s health and well-being over the course of their lives have been empirically supported (e.g., Norman et al., 2012; Jardim et al., 2018). CM has been found to be related to greater educational difficulties and hardship at school (Dovran et al., 2019), as well as to lower quality of life (Weber et al., 2016). CM is also associated with an increased risk for a variety of mental health problems such as depression, substance abuse, suicide attempts, and high-risk sexual behaviors in adulthood (Gilbert et al., 2009; Mandelli et al., 2015; Liu et al., 2017; Klumparendt et al., 2019), post-traumatic stress (Dovran et al., 2016), as well as post-traumatic stress disorder (PTSD; Vranceanu et al., 2007; Shakespeare-Finch and de Dassel, 2009). It has been shown that of all the adversities and traumas children are exposed to, CM is among the strongest predictors of PTSD (McLaughlin et al., 2017). Some of these findings can be explained by the enduring negative effects which CM has on brain development (Teicher, 2002). Taken together, these findings demonstrate the potential adverse impact of CM on psychological functioning and well-being in later life.

Alongside the impact on mental health and psychological well-being, several studies suggest that CM is related to poorer economic outcomes and to vocational vulnerability (e.g., Currie and Widom, 2010; Thielen et al., 2016). A review of the existing literature has shown that CM is related to reduced income, higher unemployment, lower level of job skill, and fewer assets in adulthood (Bunting et al., 2018). CM leading to greater financial strain during adulthood has also been found in a longitudinal study (Henry et al., 2018). Thus, CM appears to be linked to work-related vulnerability in adulthood.

Notwithstanding the potentially devastating outcomes of CM, over 25 years of research has documented the possibility of positive changes that can occur following such events, commonly referred to as post-traumatic growth (e.g., Schaefer et al., 2018).

### Post-traumatic growth

A commonly used definition of post-traumatic growth (PTG) is provided by Tedeschi and Calhoun (2004), p.1: “Significant beneficial change in cognitive and emotional life beyond previous levels of adaptation, psychological functioning, or life awareness.” This positive psychological change occurs after facing traumatic life events (Calhoun and Tedeschi, 2001). The “growth” component of this construct refers to one’s subjective perception of the benefits gained from coping with the trauma and its aftermath (Zoellner and Maercker, 2006). Three categories of positive psychological change are usually reported by trauma survivors: changes in self-perception, changes in relationships with others, and changes in philosophy of life (Tedeschi and Calhoun, 1996). Following the development and widespread use of a self-report instrument, the Post-traumatic Growth Inventory (PTGI; Tedeschi and Calhoun, 1996), many researchers have conceptualized growth in five domains: personal strength, relating to others, appreciation of life, openness to new possibilities, and spiritual change. The first three of these (personal strength, relating to others, and appreciation of life) align with the original domains; openness to new possibilities refers to newfound interests, paths, or activities that can unfold following a trauma; and spiritual change reflects an engagement with religious, spiritual, or existential matters (Maitlis, 2020).

PTG is experienced along a continuum, with some degree of growth reported by between 30 and 80% of people who have experienced trauma (Linley and Joseph, 2004). In fact, a recent review has shown that nearly 50% of individuals who suffered from traumatic experiences report moderate to high levels of post-traumatic growth (Wu et al., 2019).

PTG has been reported at the individual level after the experience of a wide range of life challenges and traumatic events, including natural disasters such as earthquakes (Muldoon et al., 2017), hurricanes (Hafstad et al., 2011), medical illness such as cancer (Ochoa Arnedo et al., 2019), or living with HIV (Rzeszutek and Gruszczyńska, 2018). Other precipitating occurrences include bereavement (Michael et al., 2013), or violence-related traumas such as sexual assault (Shakespeare-Finch and Armstrong, 2010), domestic and family violence (D’Amore et al., 2021), terror (Eze et al., 2020), as well as war and conflict (Zalta et al., 2017).

While PTG appears to be somewhat common, it is important to note that it does not eradicate the suffering, nor the damage caused by trauma. Rather, it may be a positive byproduct of the struggle to overcome the pain and hurt caused by the trauma. Survivors who report PTG do not necessarily feel less distressed (Tedeschi and Calhoun, 2004). In fact, many report that, alongside their ability to recognize psychological benefits from dealing with trauma, they are still suffering from its aftermath. Indeed, studies have shown that PTG can co-occur with psychological distress, such as Complicated Grief (Bellet et al., 2018), depression and anxiety (Li et al., 2021), and PTSD (e.g., Zhou et al., 2018). This may be especially true in relation to childhood traumas, such as CM, in which adverse consequences are well-documented (e.g., Gilbert et al., 2009; Mandelli et al., 2015; Liu et al., 2017; Klumparendt et al., 2019).

#### Post-traumatic growth after CM

As described above, child maltreatment has been found to elevate the risk of suffering from a multitude of detrimental consequences. However, such a childhood trauma may also bring about PTG. Indeed, positive changes have been reported by women who were sexually abused as children (McMillen et al., 1995; Lev-Wiesel et al., 2004; Hartley et al., 2016; Lahav et al., 2020), survivors of various types of CM (Hall et al., 2009), and adult survivors of institutional childhood abuse (Sheridan and Carr, 2020). Studies also show that PTG may take place throughout different developmental stages in the lives of survivors, such as in adolescence (e.g., Glad et al., 2013) or during young adulthood (Schaefer et al., 2018; Jankovic et al., 2022).

In a study of adults who were emotionally, physically, and/or sexually abused as children, almost all participants reported positive change in self-perception, about two-thirds reported positive change in their world philosophy, while only 20% reported positive change in their relationships (Woodward and Joseph, 2003). Alongside the previously recognized dimensions of PTG (personal strength, relating to others, appreciation of life, openness to new possibilities, and spiritual change), survivors of childhood sexual abuse have reported more categories of growth that are related more specifically to CM, such as increased ability to protect their children from abuse; increased ability to protect themselves from abuse and exploitation; and increased knowledge about sexual abuse (McMillen et al., 1995).

#### Post-traumatic growth as a meaning-making process

Despite its development after traumatic events, it should be emphasized that growth does not directly result from trauma. It evolves from the psychological struggles survivors encounter as they attempt to adapt to the traumatic event and to the distress and disruption it causes (Tedeschi and Calhoun, 2004; Muldoon et al., 2017). According to the theory of shattered assumptions, distress following child maltreatment emerges from the shattering of benevolent assumptions children initially held of themselves, others, and the world (Tedeschi et al., 1998; Janoff-Bulman, 2010). The destruction of such assumptions is accompanied by deep distress. Theorists in the field of PTG have argued that the extreme distress drives the process of re-evaluating one’s sense of self, others, and the world. Such a process represents a major discontinuity in personal identity, but also an opportunity to integrate and re-evaluate the self in light of the trauma (Tedeschi and Calhoun, 1995; Joseph and Linley, 2006).

Attaching meaning to the struggle with the aftermath of CM has been considered an important ingredient in one’s recovery (e.g., van der Westhuizen et al., 2022). Meaning in the context of trauma is explained as the ability to transform one’s view of the ordeal into a personal achievement, i.e., experiencing a sense of triumph despite the devastation caused by the trauma (Frankl, 2008). As suggested by the “meaning making model” (Park, 2010) as well as by Taylor (1983) the readjustment process following a traumatic event involves an attempt to find meaning in the experience, leading the individual to consider why the event happened and its personal significance and impact. Similarly, others have observed that successful recovery from trauma is usually supported by an increased awareness of meaning in the narrative which develops post-trauma (Foa and Rothbaum, 1998). In the words of Victor Frankl, a Holocaust survivor, in his *Man’s Search for Meaning*, first published in 1946 “In some ways suffering ceases to be suffering at the moment it finds a meaning.”

While meaning-making can happen in any life context, one of the main sources of meaning in modern lives is work. Today, in Western societies, in which individuals have some degree of choice regarding their occupation or workplace, work serves as a main vehicle to make sense of the self and the world (Rosso et al., 2010). Work provides the individual with an opportunity to express core values and personal strengths, to be productive, creative, and valuable and to relate to a significant group or community of other people (Lysova et al., 2018). As such, work may offer individuals in the aftermath of personal trauma an opportunity to better understand and make sense of what happened, to relate positively to others, and to be active in reconstructing their lives as agents of change. Nevertheless, empirical studies on vocational aspects of post-traumatic growth among survivors have remained relatively scarce.

#### Post-traumatic growth at work

Globally, most adults spend much of their waking hours at work (Jain and Leka, 2019). Moreover, work, like love, has been posited by many, including Freud (1963), as an adaptive vehicle in the face of existential concerns. While work provides many social and economic benefits, it also exposes individuals to occupational stressors. Specifically, working in settings in which individuals encounter trauma (either directly or vicariously) may lead to experiencing distress, and possibly Post Traumatic Stress Disorder, PTSD (Skogstad et al., 2013; Coenen and van der Molen, 2021). However, encountering such traumatic experiences at work may also cultivate positive transformation, such as PTG. In fact, workplace-related PTSD and workplace PTG are not mutually exclusive; rather, they usually co-exist (Cohen and Collens, 2013; Finstad et al., 2021).

Previous research regarding workplace PTG has focused mainly on several distinct types of work-related trauma. First, most studies have explored workplace PTG in job contexts vulnerable to direct trauma exposure, such as military, police, firefighting, and medical settings (Maitlis, 2020; Olson et al., 2020; Finstad et al., 2021). A second branch of studies has explored the impact of vicarious exposure created by working with traumatized populations, including various negative effects as well as PTG (Cohen and Collens, 2013). Recently, studies have also examined PTG in the context of a shared traumatic reality (Baum, 2014), i.e., when professionals experience the same traumatic event or series of events alongside their clients (Finklestein and Laufer, 2021; Dahan et al., 2022).

Notwithstanding the contribution of previous studies on work-related PTG, the existing literature has certain limitations. To the best of our knowledge, the aforementioned studies have explored work-related trauma, i.e., trauma which happens at the workplace itself or that the workers are exposed to there. However, in the light of the reviewed literature, work-related PTG may also occur as a result of a personal trauma, rather than a work related one. Moreover, positive transformative changes at work may result from the struggle to heal from CM and may evolve through meaning-making processes.

## Research question

By focusing on the underexamined context of vocational lives of survivors, the current exploratory qualitative study attempts to shed light on the following question: What is the lived experience of work-related post-traumatic growth among high-functioning adult survivors of CM?

## Materials and methods

We adopted a phenomenological approach, which focuses on exploring the meaning of phenomena in human experience from the perspective of the individuals themselves (Giorgi, 1975). We aimed to listen closely to the participants’ lived experiences and their construction of reality through narration (Holt and Sandberg, 2011; Corbin and Strauss, 2014). A qualitative exploratory study was chosen due to several reasons. First, some scholars claim that it allows for greater in-depth exploration of novel research topics and phenomena than quantitative designs (Wilhelmy and Köhler, 2022). Second, it is particularly useful in areas of research where there is limited empirical evidence or theory to guide subsequent quantitative research (Cassell and Symon, 2011). Third, it allows researchers to ask questions about sensitive topics (Kaplowitz and Hoehn, 2001). Lastly, qualitative research is also used for the purpose of elaborating on theory. Researchers adopting such approach are familiar with the existing literature and design the interview study to build on previous work (Dunwoodie et al., 2022). This study was part of a larger research project exploring the vocational experiences of working adults who were maltreated as children.

### Recruitment

Recruitment for the study focused on high functioning, working adults who had been abused or neglected as children. The main rationale for focusing on high-functioning survivors was twofold. First, due to the scarcity of research on work-related PTG among CM survivors, an effort was made to recruit a sample who might report such growth. Second, since many CM survivors cope with adverse consequences, we chose a less vulnerable sample for this early stage in the research. We intentionally targeted survivors who could provide detailed and rich descriptions of their vocational lives. A previous study exploring work experiences of CM survivors was used to determine inclusion and exclusion criteria (Thomas and Hall, 2008). The criteria that we used for inclusion were that the prospective participants identify themselves as survivors of neglect or child abuse; that they be working full time; that they had remained in the same jobs over the past 3 years (employment stability); and that their annual income from their employment be average or above. The criteria for exclusion from the study were if individuals were currently involved in interpersonal violence; currently exhibiting symptoms of psychosis, severe depression, or suicidal ideation; and currently abusing alcohol or drugs. We screened the prospective participants in a preliminary conversation by phone where they were asked to reply to questions probing these matters. Only those who claimed to be meeting all the inclusion criteria without meeting any of the exclusion criteria were asked to take part in the study.

Because of the topic’s sensitivity, convenience sampling was used *via* three primary means of recruitment. Convenience sampling is a type of nonrandom or nonprobability sampling where members of the target population that meet certain practical criteria, such as easy accessibility, availability at a given time, or the willingness to participate are included for the purpose of the study (Etikan et al., 2016). We began by contacting personal and professional contacts, asking them to refer potential participants who might be willing to take part in the study. Such contacts were colleagues from academia and from the field, former graduate students who have completed their studies, personal acquaintances such as people who studied with the author/s towards an academic degree, members of professional forums that the authors belong to, etc. We were able to identify 13 prospective interviewees through this means, of whom 12 agreed to join the project. Our second step was to screen articles in the local and national press and reach out to individuals who had made public their experience of childhood maltreatment. This enabled us to find five more participants for the study. Our third step was to utilize snowball sampling. At the end of an interview, we asked individuals whether they could recommend anyone who was qualified to join the study by meeting its criteria. This strategy enabled us to recruit three additional participants. We built the sample “one at a time,” attempting to assure that both binary genders were represented, as well as a variety of professions and occupations, disparate socioeconomic childhood backgrounds, and experiences of different kinds of CM.

Sample size was determined by the saturation principle: data were collected and analyzed until no new themes emerged and the addition of participants did not offer any new insights into the research questions (Corbin and Strauss, 2014; Dunwoodie et al., 2022).

### Sample

There were 20 high-functioning adults in our sample, which was composed of 13 women and 7 men between the ages of 28 and 60. Regarding CM, 75% stated that they had experienced emotional abuse, 50% physical abuse, 55% neglect, 35% sexual abuse, and 40% verbal abuse. There were 79% of the participants who stated that they had been the victims of more than one kind of abuse. All the members of our sample described their experiences of CM as prolonged, lasting at least several years, and severe in nature. Due in most cases to maltreatment, slightly more than a third of the participants (37%) were placed in an out-of-home residential care setting during their childhood or adolescence. Although the childhood backgrounds of our participants differed from a socioeconomic standpoint, most had achieved a high level of education and were currently white-collar professionals (e.g., lawyers, therapists, and school principals) with middle to high income and social status. The sample was well educated, as 70% of the interviewees held either a master’s or a doctorate degree. Half of the sample were currently in managerial positions. The majority were involved in a committed relationship (79%) and had children of their own (84%). See Supplementary Tables 1, 2 in the Supplementary materials for a more detailed background of the study participants.

### Procedure

A time and place for the interview were selected by each of the individual participants. The interviews, which lasted between one and a half and 2 h, were conducted face-to-face, and were structured and in-depth. All participants signed an informed consent agreement, and their confidentiality was ensured throughout the study. The Human Subjects Research Committee of the academic institute where the second author works provided ethical approval. A team of four undergraduate psychology students who were trained research assistants audiotaped each interview and then transcribed it verbatim.

Following the guidelines suggested by Dunwoodie et al. (2022), the interview protocol was developed so that questions were open-ended and broad enough to enable participants to talk freely about their work experiences, and to allow for rich data generation. We started each interview with an invitation to openly describe current work position and vocational history from the time of their first employment. Next participants were invited to share their perspectives regarding work relationships with supervisors and colleagues, their occupational choices, and what their work meant to them. These broad questions were followed by probes to encourage elaboration of responses. Special attention was given throughout the interviews to expressions of self-reflection regarding work-related positive change and growth. After examining current and past vocational experiences, participants were asked to describe their childhood (incidents of CM were reported at this stage), followed by a question on how they saw their vocational future. At the end of the interview, we posed a summary question to the participants on how they viewed a possible connection between CM and their work history. The full interview protocol is described in the Supplementary materials. (We have not explored all the topics mentioned above in the current manuscript.) Demographic details were also collected.

### Data analysis

Data analysis was based on guidelines for phenomenological analysis, as suggested by Hycner (1985). After transcribing the interviews, the authors acquired familiarity with the data by rereading the interviews several times until immersion, allowing them to unfold with as little interpretation as possible.

The next step aimed to develop a sense of the whole, noting general impressions and specific topics of interest, in preparation for delineating units of general meaning. In the following step, the authors independently and systematically retained data from words, phrases, paragraphs, and significant nonverbal communication reflecting work-related PTG processes. In addition, textual units were retained referring to childhood abuse and neglect, enabling categorization of type and extent of maltreatment for each participant. Maltreatment type was categorized according to the conceptualization offered by the ACE (Adverse Childhood Experiences) study (Dube et al., 2001). The overall process resulted in a dataset of general meaning units referring to work-related post-traumatic positive changes, as well as childhood maltreatment.

Next, units of meaning relevant to the research question were delineated, focusing on experiences reflecting specific types of work-related growth. These meaning units were then used to create descriptive categories of basic themes and thus construct an initial framework for further analysis. At this stage, the themes were grouped across the interviews into clusters of similar issues, from which the final main categories of the model emerged. The research team met regularly, addressing each unit of general meaning. In cases of discrepancies, the team contextualized excerpts based upon the original interviews and discussed optional meanings until resolution (Denzin, 2017).

Subsequently, the first author iteratively examined and cross-checked data coding by the second author, and vice versa, to verify units of relevant meaning. Redundancies were pinpointed and removed, and themes were modified as deemed necessary. The next step entailed identifying commonalities, so that units of relevant meaning could be clustered into themes while still preserving individual variation, as variations may indicate the theme’s significance (Hycner, 1985). For example, descriptions of occupational strengths and of personal resistance were revealed as two specific categories reflecting the theme of self-related PTG. By the end of this stage of the data analysis, five types of PTG emerged.

Following Sanders’ (1982) phenomenological approach of open coding as an inductive process, we did not use *a priori* categories, although we kept in mind Tedeschi and Calhoun’s (1996) conceptualization, and eventually matched the types of positive changes which emerged in the study.

## Results

**Figure 1:**
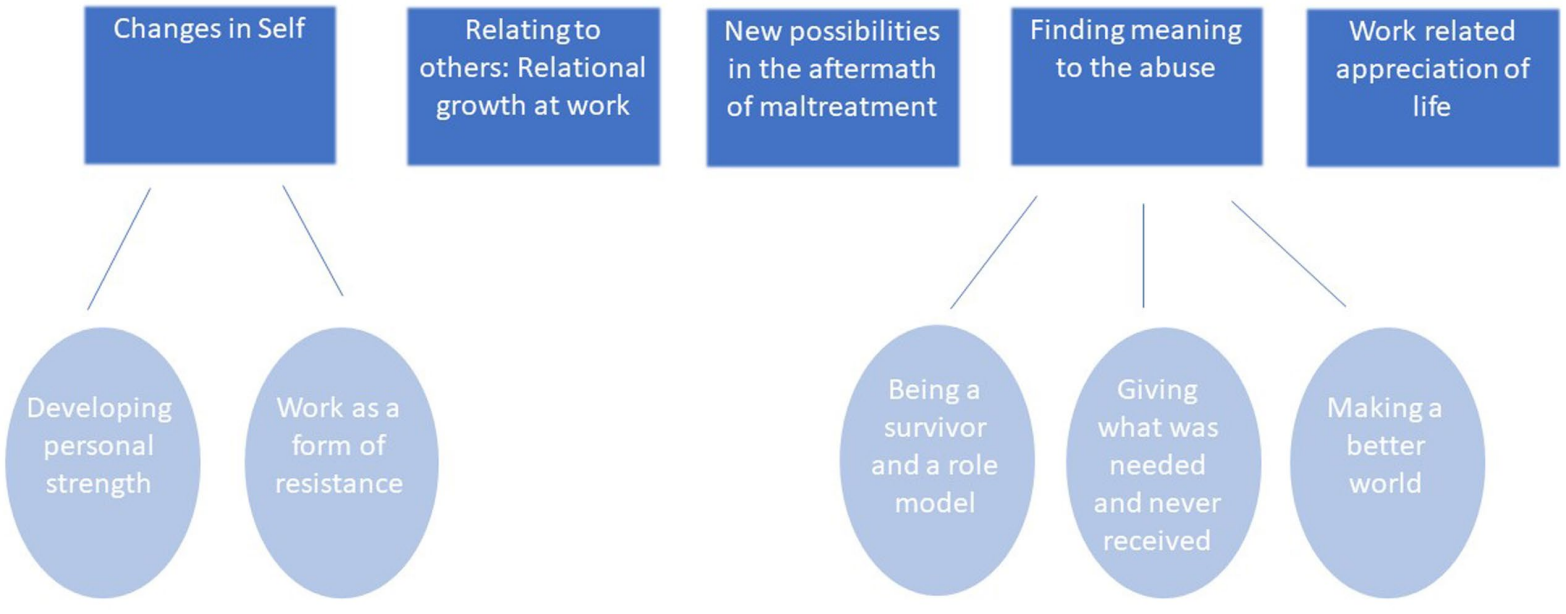
Five domains of work-related post-traumatic growth as described by participants.

Findings revealed rich descriptions of work-related post-traumatic growth, regarding changes in the following domains: (1) Self (developing personal strength and making work a form of resistance); (2) Relating to others—relational growth at work; (3) New possibilities in the aftermath of maltreatment; (4) Finding meaning to the abuse (being a survivor and a role model, giving what was needed and never received, making a better world); and (5) Work-related appreciation of life (see Figure 1). Although the analysis was not limited to the domains suggested by Tedeschi and Calhoun (1996), eventually the themes which emerged seemed to correspond well with these pre-existing domains of PTG (personal strength, relating to others, appreciation of life, openness to new possibilities, and spiritual change), while subthemes seem to offer some elaboration of the original model. All names of interviewees are pseudonyms.

### Changes in self

#### Developing personal strength

Regarding changes in self, participants recognized and gave voice to the inner strengths that resulted from their struggle to heal from traumatic pasts and described them as highly present in their vocational lives.

Yolanda, 44, who underwent neglect, and both emotional and sexual abuse in childhood, reflected on how she functioned as a school vice principal, working with female adolescent students:

“The question is, and I sometimes ask myself this, do you have to go through what I went through in order to be smart about these things, or is it possible without it (sad laughter…)? I do not know … It’s very funny, but I am almost glad about what happened to me, since looking back… I am a much better person, totally so! I am a thousand times better and I can help a thousand times more, but I had to undergo an awful, painful journey until the age of 21…”.

Yolanda, as many other participants, proceeded to talk about the unique abilities and insights she has to offer, as a survivor, to students undergoing challenging traumatic life experiences.

#### Work as a form of resistance

The strengths and career successes experienced by survivors were often described as forms of resistance to the traumas inflicted by maltreating parents.

For example, Nina, a 50-year-old coach, who works with senior managers in organizations, underwent emotional and verbal abuse in childhood. Nina described that she feels as if both her height (being a very tall woman), as well as her vocational choices, are a form of resistance to her parents’ upbringing:

I tell myself that my DNA objected to everything I heard, and all I was told. I was told to shut up, I was told I was small and insignificant, and that nothing I said matters. And I turned into someone that is listened to by very senior managers in the country, I am their confidante, and they confide in me … It is like this huge objection to this message that I was small and did not know …. You can see that … all the time … I did not believe it. I refused to accept it and to believe it.

Nina suggested that by rejecting the hurtful messages conveyed by her parents, she was able to protect herself and to later develop a positive work identity, countering early familial experiences.

While Nina savored the experience of being listened to by highly influential managers in her position as a coach, other participants became such managers themselves. Ian, age 37, a municipal politician, who reported childhood emotional and physical abuse and neglect, and has worked in management positions from the beginning of his career stated: “I can explain to you my yearning for leadership … My father told me that I was nothing and locked me in the closet; he said I was worthless, so I set out to prove … that I am worthy.” Later in the interview, he added: “I came from death, really, from death, and somewhere along the way when I chose to live, I said that one day I will run this country. I will change things.” Hence, Ian connected his work-related strengths and ambitions, especially leadership related ones, to his childhood trauma. For him, turning into a man who has influence over others is the vehicle of resistance to the toxic messages regarding worthlessness his father conveyed to him.

Other participants’ resistance was expressed by a dialog with the vocational future predicted to them by abusive parents, sometimes as a form of verbal abuse. Michelle, a life sciences researcher, age 39, who reported childhood neglect, as well as emotional, physical, and sexual abuse, described a painful incident from her childhood, which she considered to be a defining event. After experiencing sexual assault from other boys in her neighborhood, she was offered no support by her abusive parents, but rather, she was blamed and attacked by her own father:

And then we came home and he pushed me into the shower, with all my clothes on, and he tried to rip my shirt off and started hitting me and said: "I know what you'll be when you grow up, you'll be a prostitute!" and … (Sighs…). That sentence stayed with me for a long time, maybe it is the reason I became a Doctor of Biology, just to prove to him that I didn't [turn out to be a prostitute], and that's it ….

Thus, by creating a meaningful dialog, participants negate the hurtful messages of maltreating parents, and cultivate an alternative self. Instead of being worthless, small, and insignificant, their workplace experiences help to transform them into what they perceived as influential, successful, worthy adults.

### Relating to others: Relational growth at work

In the second identified pattern of PTG, participants described how their relationships at work start out as challenging and as possibly reenacting negative childhood experiences. Over time, however, they undergo positive changes in relating to others in the workplace, both supervisors, as well as colleagues.

Tara, age 44, a teacher who underwent childhood neglect stated:

It is hard for me to acknowledge, I realize, that the fact that I did not have a good relationship with my mother affects my relationship with other people, and especially authority figures. I find myself mad at them and fighting them, because this is what I know, and I am doing a lot of work on myself on this issue. With my previous manager, I had an extremely good relationship … I was 24 years old, and a very young teacher and she really helped me grow, by providing me with both tools and support.

Tara directly linked her relational difficulties with authority figures at work to her relationship with her neglectful mother, but she also recognized that supportive workplace supervisors can help create positive changes in this regard.

Positive changes were described not only in the context of relationships with workplace supervisors, but also regarding relationships with colleagues. For example, Nina compared her relationship with her previous business partner and the current one:

I feel I have learned a huge lesson about setting boundaries with people, you know. It's this place of emotional extortion that once I did not know what to do with, and at a certain point in time, I had to say goodbye, so today it is about setting boundaries, yes, really setting boundaries.

It is not surprising that setting boundaries arise as a central issue in Nina’s experiences, as homes where maltreatment takes place often challenge the ability to set proper relational boundaries. To sum up, the second theme reveals relational growth processes in the workplace, with various figures, both supervisors and colleagues.

### New possibilities in the aftermath of maltreatment

In the third domain, participants described how they perceived vocational settings as offering new opportunities for work-related growth.

Mona, age 44, who underwent emotional abuse and neglect, had begun an occupational change a short time prior to the interview. She had been a freelance graphic designer for most of her adult life and had recently shifted to becoming a teacher. This shift offered a new opportunity, which she embraced with ambivalence, as she described:

For many years I felt damaged, and the opportunity to work with people and to be their teacher, suddenly I must let go of the damaged self, and emotionally I am not there yet. You see, I do not trust myself completely. You know, there are complex things that … affect. I need to, you know … Suddenly I find myself a teacher after so many years that I sat behind the screen and did not need to deal with the world. I was disconnected from the world for almost 15 years ….

Mona fully realized that the new position could help her let go of what she saw as a “damaged self.” However, she was uncertain whether she was ready for such positive change to occur.

Ian experienced such an opportunity as an adolescent, on his first job, living away from his maltreating home:

I started to work at the kibbutz once a week, at the henhouse. That is where I found meaning … and then [after some time] they let me run the place, manage it! So, at 17 or 18, I ran the henhouse for eight months, so you tell yourself-I am worth something. I have something to do, I am valuable!

Had Ian not been removed from his home by Child Protective Services as a child due to maltreatment, he might not have had the opportunity to work at a young age with farm animals and care for their needs. This experience undoubtedly left an impact on him and perhaps directed him to a career in management. Whether in early career stages or in mid-career, both Ian and Mona’s words show how their work lives offered new possibilities that helped them address their childhood traumas.

### Finding meaning to the abuse

Participants in this study seemed to be especially engaged with a search for meaning, fueled by their childhood maltreatment, to which the workplace managed to provide some answers, as will be portrayed in the subthemes below. Three subthemes will be presented: being a survivor and a role model; giving what was needed and never received; and making a better world.

#### Being a survivor and a role model

The first work-related positive change in this subtheme stems from the opportunity to serve as a role model to fellow survivors in the workplace. Sophie, age 46, an attorney, underwent emotional and physical abuse, as well as prolonged paternal incest starting at a young age. As an attorney, she helps many clients who share similar backgrounds in terms of maltreatment:

I want … to set an example, be a role model to so many girls who have been maltreated … So, they will know that I was like them, I was poor and had nothing to eat, and as a student I cleaned and scrubbed soot off of warehouses after fires and that I was raped and that I … I went through the most difficult things and I decided that I will not give in … And I am simply living my dream now and I insist, I want everybody to know where I came from, because when these girls know where I came from …. it works.

Sophie’s words demonstrate the opportunity to grow out of her identity as a victim/survivor, into that of a role model to others. Espousing a newfound philosophy of life, which guides their behavior, survivors serve as role models at their jobs. They model what it means to undergo such traumatic experiences, as well as the resilience and growth that can emerge from it.

#### Giving what was needed and never received

A second subtheme refers to finding meaning through working with children and adolescents who are undergoing similar experiences to the ones undergone by the participants. However, unlike in the previous suggested subtheme, participants are not serving as role models, but providing the help that they themselves would have benefitted from as children, had it been available. Whether the similarity is in feelings or experiences, it seems that the workplace offers participants a way to find meaning regarding their childhood maltreatment *via* relationships that reduce other people’s suffering. In a way, it is about giving to others what they themselves needed but never received.

Naomi, a social worker who grew up in a family of ten children, was sexually abused by her father as well as by a brother. At the time of the interview, she was starting a new position, working with families of maltreated children in their homes. She was asked by the interviewer about her feelings towards her new position and she replied:

Oh, that is a [good] question. I am very excited about this job because for me, if only there had been a social worker in my home, if only there was someone who saw me up-close, if only I could have been seen …. So, for me going to the homes and seeing the parents as well as the children, it is sort of a gift …

Neal, age 50, a school vice principal, teacher, and therapist who underwent physical abuse and neglect in childhood, shared a similar message:

I think that what I felt, I guess a lot of adolescents feel this way, but I felt as if I am the loneliest person in the world, I mean I was certain that there is nobody out there like me, none. None of the adults would talk to me, it was this huge, huge sense of loneliness. And I have worked with many children and adolescents and what is common to all of them, from what I see, is that they are yearning for relationships, and they are yearning for adults to listen to them… This is what drives me crazy, it always surprises me and makes me emotional.

Taking it one step further, Sophie, an attorney who specializes in representation of female victims of sexual violence, and who is a survivor of prolonged paternal incest, reflected on her career choice: “I always go to these places. Women, women, prostitution, women, women, women, I wonder why (chuckle). I am drawn to this place, to rescue, rescue the girls, every one of them ……”

She continued to describe her complex emotions and internal dialog with the maltreated girl she used to be:

I have a few "Sophies" inside me … Inside me there is Sophie the little girl who was maltreated and is stuck at the age of 12, this girl who was hurt, she just … I carry her in my arms, her dead corpse, I carry her everywhere. I have a lot of anger towards that girl, a lot of pity, all sorts of emotions, but it is also a choice that she dies and there will be a new Sophie, because I cannot be her, I cannot be her … I chose to detach …

Finally, Sophie connected her painful history to her daily work by saying: “Every girl like that which I manage to help, … I am helping the girl that was me, I am just helping her, it is in her honor, yes, exactly.” Sophie finds meaning in the abuse she suffered by offering help to other girls, help which she felt was not available to her.

Moving from present work experiences to future career plans, Yolanda described how she planned to advance from being vice principal to principal in a high school for girls, a workplace which provided her with an opportunity to offer the students and their families what she was missing when she was growing up:

I should be a school principal, and especially with adolescent girls. It is definitely because I had such a horrible time during adolescence, so appalling, I was depressed for years and so I know I have something to contribute to these girls, as well as to the adults who are in contact with them, explaining things to them, giving them information…

Yolanda hoped to give to others what she felt she never received, much like Sophie, Neal, Naomi, and other participants in the study. Their wish to give seems to be part of their existential journey and offers them another path to find meaning in the horrors of their childhoods.

#### Making a better world

A third subtheme refers to finding meaning through work by attempting to make the world better. This search was evident through all the stages of the participants’ careers, taking on various forms. Some described having such dreams early on, as adolescents. For example, when having to choose a field of study in high school, Michelle selected biology, a commitment that remained steady during her undergraduate and graduate studies, as well as her *post doc*. Michelle explained her choice: “The fantasy was to save someone. If I cannot save myself, then at least I will save something in the world.” Indeed, later in her career, she studied cancer in the hopes of contributing to the discovery of a cure for it.

Describing her adolescence, Nina recalled:

Look, when I was eighteen, I had this yellow notebook and, in that notebook, I wrote that my vision was to end human suffering. I always laugh when I talk about this in workshops, the aspirations that an eighteen-year-old girl has, probably out of my own suffering, but not only from that … But I really do feel that this vision … I wrote it down, it is there, and it has stayed with me along the way. I mean, I really feel that this is my mission, to always feel that you are making a change, making a difference, that somehow the suffering in the world decreases.

At the age of 44, Mona reflected on her many years of work as a graphic designer:

I look at my own biography, how during those years [childhood] the world was ugly and unjust. In a way, I am always creating composites. What do I do at my work? I take elements and I make composites. I organize things in an aesthetic manner, and I think this is because of the hole that was created inside of me. My sense of aesthetics and art has been very strong from a very young age, so I want the world to be prettier. I keep organizing things to make them prettier and that is what I have been doing throughout the years.

As Michelle and Mona described, the world they experienced as children needed to be fixed, as it was unjust, cruel, ugly, and filled with suffering. The participants appeared to be searching for ways to change this world for the better. Even if this may appear to be a grandiose motivation, it seems that it serves as a way to create meaning out of their own childhood traumas. Later in their careers, such ambitions became concrete career decisions, as described by Sophie, an attorney:

Each case that I take must fit two criteria. First, whether I can get the client money. And at the same time, it must be a case which will bring about social change. I only take cases in which the ruling can create a systemic widespread change for many people and that is what I do.

Indeed, the impulse of striving to make a better world was inherent in many of the work narratives brought forward by the participants of the study.

### Work-related appreciation of life

A fifth theme of positive changes refers to a sense of work-related appreciation of life. This was reflected in the words of Sophie:

I am 46 years old and I think that this is the time in my life that I am working the hardest I have ever worked… But this is the time in my life where I am not only working the hardest; I am also the happiest and most satisfied. Really. I wake up every morning and I feel the joy of creation.

As similarly described by other participants, Sophie found ways in which her career provided her with numerous growth-related opportunities. It is not surprising that she reported such appreciation of life and gratitude. Work offered participants an opportunity to acknowledge the positive aspects in their lives and paradoxically, to provide a sense of reconciliation with painful childhood experiences.

## Discussion

Findings of the study indicate that participants’ work-related PTG could be categorized by using the five domains of growth (personal strength, relating to others, appreciation of life, openness to new possibilities, and spiritual change) suggested by Tedeschi and Calhoun (1996). However, these themes were uniquely linked to the context of their vocational lives, and several subthemes which emerged offer an extension of these domains. Participants directly connected these positive workplace changes to their past as maltreated children. The findings regarding developments in the following domains will be discussed below: changes in self, relating to others, new possibilities, finding meaning to the abuse, and appreciation of life.

Previous studies have documented positive changes regarding self-worth in the aftermath of trauma. In a similar vein, the current study shows that struggling with the consequences of CM, while it poses many difficulties, also leads to developing strengths which later manifest themselves in work settings. This is especially important since trauma usually harms the views regarding the self (Janoff-Bulman, 2010). These findings show that participants grasped the changes they underwent as dialectic in nature: alongside the benefits they perceived, they also reported great suffering and challenges related to self-perception.

Another form of positive changes in self, which emerged from the interviews, was grasping work as a form of resistance to the hurtful and demeaning childhood messages given by parents. Participants described their career choices and activities as a form of opposition to such belittling messages. This growth may be based, first and foremost, on refusing to internalize the toxic messages they received as children. Later, as adults, this subtheme was framed as a continuous dialog with the maltreating parent. In their eyes, this internal dialog takes place throughout their careers. Many of their successes and achievements are experienced as a celebration, which serves to prove the maltreating parent wrong.

In terms of relational change, previous research has shown that adult survivors of CM may struggle with general relational difficulties (e.g., Paradis and Boucher, 2010), as well as relational challenges at work (Lisak and Luster, 1994; Hall, 2000; Ballou et al., 2015). For example, survivors may reenact maladaptive internal schemas (Ballou et al., 2015) or suffer from workplace revictimization (Icekson et al., 2021). However, the work environment may also offer opportunities to experience growth *via* workplace relationships.

Regarding relational growth, while challenges were not infrequent in the narratives of survivors, our findings suggest that participants also experienced positive relational changes throughout their careers, with both colleagues and supervisors. Specifically, the participants told of an improved ability to set boundaries, as well as to benefitting from a caring supervisor. Coping with traumatic events offers survivors an opportunity to reevaluate their relationship with others, to develop new relationships, and to establish more intimacy in existing relationships (e.g., Weiss, 2004; Canevello et al., 2016). While previous studies on survivors of CM have mainly focused on relationships with friends and family members (e.g., Glad et al., 2013; Schaefer et al., 2018), this study extends previous findings through suggesting a path of work-related relational growth. Moreover, since survivors are coping with long-term effects of aversive childhood relationships, the growth-related patterns which were reported correspond with the characteristics of these relationships. Growing up in chaotic and unpredictable homes, in which personal boundaries of the child were not respected, the increased ability to set boundaries is especially noteworthy. Our findings are in some respects congruent with the findings of McMillen et al. (1995), according to which survivors of CM felt better able to protect themselves from abuse and harm.

Theoretically, such findings may be explained *via* changes in psychological constructs such as attachment style (Bowlby, 1982), or differentiation of self (Bowen, 1985). According to these theories, significant relationships during adulthood (including workplace relationships with supervisors) not only resemble primary internal working models, but also could alter them (e.g., Blustein, 2011; Mikulincer and Shaver, 2020). Though our findings offer an important perspective, they should be considered with caution. The relational growth paths for adult survivors of CM in the workplace, as well as their underlying theoretical mechanisms, have been underexplored and require further scholarly attention. Moreover, this domain of growth has previously been found to be less common among survivors (Woodward and Joseph, 2003), since in many instances the trauma persists in relational issues (e.g., Paradis and Boucher, 2010).

Turning to another domain of change, our participants experienced new opportunities *via* the workplace. The survivors told of exploring new vocational opportunities which opened as a result of the struggle to cope with CM. These new opportunities not only offered them a chance to earn a living, but also to explore their strengths, and to experience themselves as competent, able, and responsible. As has been suggested by Tedeschi and Calhoun (1996, 2004), during the process of struggling with adversity, survivors discover new options and opportunities that were not available to them prior to the trauma.

The opportunities that were pursued by our participants entailed actual, “real-life” career changes, similarly to what has been referred to as “real” growth by Frazier et al. (2001). Such changes correspond with one type of change offered by the Janus-Faced model of PTG (Maercker and Zoellner, 2004). According to this model, PTG entails two components. These components might be found in the same individual or within different individuals. Each of them has a different developmental path and different influences on adjustment. The first is a functional component which represents authentic growth and is related to healthy adaptation. The second is a dysfunctional one which strengthens an illusion and might co-exist with denial. Such dysfunctional aspects of illusory PTG have also been empirically supported (e.g., Lahav et al., 2020). However, the narratives brought forward by our participants appear to tell of the presence of the functional component, as the opportunities opened new paths for them. Indeed, the model suggests that in positive adjustment, the functional component grows over time while the dysfunctional one subsides.

Not surprisingly, survivors’ attempts *via* work to give meaning to the abuse they experienced emerged as a substantial part of their narratives. Within this theme, three subthemes emerged: being a survivor and a role model, giving what was needed and never received, and finally, making a better world. This corresponds with the domain of spiritual change, a feature of PTG which reflects an engagement with spiritual, religious, or existential matters (Tedeschi and Calhoun, 1996). Participants in this study seemed to be especially engaged in an existential search, fueled by their childhood maltreatment, to which the workplace managed to give some answers.

It has been previously claimed that recovery after trauma involves the alteration of one’s meaning structures (Linley and Joseph, 2004). When trauma disrupts the identity of the self, as it frequently does, it also disrupts associated motivations and meaning in life (Muldoon et al., 2019). According to Tedeschi and Calhoun (2004) meaning-making is a core component of reframing the traumatic experience. Indeed, the positive relationship between PTG and meaning-making is evident in the literature (Janoff-Bulman, 2006; Triplett et al., 2012; Walker-Williams and Fouché, 2017; Zeligman et al., 2019).

Findings from several reviews corroborate that PTG after childhood trauma can be understood as a meaning-making process. In a literature review regarding PTG after sexual violence, findings indicated that survivors reported positive change characterized by engaging in advocacy and activism as a concern for others (Ulloa et al., 2016). Also, a recent systematic review of PTG after sexual traumatization found that utilizing altruistic actions and activism to prevent further sexual victimization facilitated growth among women (Guggisberg et al., 2021). Finally, a recent review of meaning-making by women survivors of childhood sexual abuse found that advocating for and tending to those who have been in similar situations, helps create a post-trauma identity by acknowledging the strengths born from the struggle to cope with CM (van der Westhuizen et al., 2022). Taken together, these reviews indicate that helping others may be a therapeutic vehicle for PTG, corresponding well with the meaning-making journey described by our participants.

The participants in our study seemed to direct their altruism and dedication to others *via* their work lives. Such a perception regarding work corresponds with the view of work as a calling. It has been suggested that the two strongest characteristics of this concept are a powerful sense of the meaningfulness of one’s career and the feeling that it is being used to help others in some fashion (Dik and Duffy, 2009). These two dimensions were highly evident in the stories shared by many participants. Some have suggested that the choice of a vocation as a calling is guided by a transcendent force (Hagmaier and Abele, 2012).

Lastly, as participants in the study reflected on their lives, they expressed a sense of work-related appreciation, gratitude, and fulfillment from their careers. Child maltreatment threatens the sense of self and many of our participants told of an ongoing dialog with death and dying. Such proximity with traumatic experiences and the threat of death from a young age undoubtedly creates an existential quest. This may lead to a sense of despair, but also to an ongoing struggle to develop an appreciation of life. Work seemed to offer our participants a means to experience life as positive and valuable, as has been described in the various themes discussed above. Hence it is not surprising that, overall, the sense of work-related appreciation of life is also positively enhanced.

To sum, while the vocational arena may entail many possible struggles for survivors of CM (e.g., Currie and Widom, 2010; Thielen et al., 2016), the results from this sample of high-functioning adults show, that for some survivors of CM, work can be a source of positive psychological changes, if they are able to not succumb to the challenges involved in it. Participants in the current study not only had impressive and successful careers, but they also gained many opportunities to heal some of their long-lasting psychological wounds.

## Limitations and future research

As a qualitative study, several inherent limitations should be noted. The first potential limitation is interviewer bias (Amis and Silk, 2008), which we attempted to minimize by following rigorous phenomenological inquiry guidelines (Tracy, 2010). Second, sample bias may influence the results, and the sample of high-functioning adults may be an overly homogeneous one in terms of current socioeconomic status, education level, as well as openness regarding past CM. Future research should incorporate more varied samples in terms of socioeconomic status, ethnicity, and work status, as well as take into consideration past PTSD, history of treatment, the severity of abuse, and level of education when aiming to explain work-related PTG. Future samples may also offer an opportunity to explore whether certain themes may be more frequent among certain types of professions. Moreover, our findings are based on a relatively small sample, and may be influenced by social desirability or memory bias, especially for those participants who were recruited through acquaintances. Understanding PTG should consider its developmental nature, and thus, we recommend that future studies on work-related PTG employ quantitative methods, as well as longitudinal designs. In addition, further studies should consider the possible influence of demographic, organizational or abuse-related variables on PTG. Additionally, due to the study design, it is not possible to determine whether the positive changes described by participants can be strictly considered PTG, as some of these changes (e.g., learning to attain better boundaries or finding a new vocational path) may be considered part of normative adult development. Extending the methodological approach to studying the vocational lives of workers who are CM survivors and those who are not, could help to discern the concepts suggested in the current study. Therefore, despite their contribution, the current findings should be interpreted with caution.

### Conclusion

Over the past 20 years, research has certainly shown the existence of post-traumatic growth (PTG) following life-threatening traumatic experiences. Positive psychological changes have been observed in relation to various types of traumas and have been documented across cultures and populations. However, work-related PTG has been less explored. Previous studies have demonstrated that work-related PTG may be apparent when workers are exposed to traumatic experiences at work, either directly or vicariously. The current study contributes to this line of research, by demonstrating the existence of work-related PTG stemming from trauma occurring in childhood, specifically childhood maltreatment.

All five previously recognized domains are apparent in the narratives of participants, when describing work-related PTG, i.e., personal strength, relating to others, appreciation of life, openness to new possibilities, and spiritual change. Moreover, the vocational experiences described by participants portray a meaning-making journey in their work lives offering resistance to demeaning childhood messages and helping to develop personal strengths. It also offers an opportunity to experience beneficial relationships both with managers and colleagues, as well as opens more opportunities for further personal and professional development. Through their existential journies, survivors derive meaning from being role models, giving others what they themselves have never received, and striving to make a better world. Finally, participants describe greater appreciation of life through their work. To conclude, the workplace seems to be more than a location to make a living for some survivors of CM, but rather an arena that holds many possible answers to their lifelong quest to make peace with their wounded inner children.

## Data availability statement

The datasets generated during and/or analyzed during the current study are not publicly available due to ethical standards. Requests to access the datasets should be directed to avitalk@ruppin.ac.il.

## Ethics statement

The studies involving human participants were reviewed and approved by Ben-Gurion University of the Negev, Beer-Sheva, Israel. The participants provided their written informed consent to participate in this study.

## Author contributions

AK-T and TI recruited and interviewed participants, performed the qualitative analyses, and jointly contributed to the development of the study design, integration of findings, and writing of the manuscript. All authors contributed to the article and approved the submitted version.

## Funding

This research was supported by funding from Ruppin Academic Center, Peres Academic Center, and the Haruv Institute. These organizations had no involvement in the study design; in the collection, analysis, and interpretation of data; in the writing of the report; and in the decision to submit the article for publication.

## Conflict of interest

The authors declare that the research was conducted in the absence of any commercial or financial relationships that could be construed as a potential conflict of interest.

## Publisher’s note

All claims expressed in this article are solely those of the authors and do not necessarily represent those of their affiliated organizations, or those of the publisher, the editors and the reviewers. Any product that may be evaluated in this article, or claim that may be made by its manufacturer, is not guaranteed or endorsed by the publisher.
