# Supplementary material for: A phenomenological exploration of work-related post-traumatic growth among high-functioning adults maltreated as children
Source: Front Psychol. 2022 Dec 22;13:1048295. doi: 10.3389/fpsyg.2022.1048295 (PMC9814126; doi:10.3389/fpsyg.2022.1048295)
Supplement: Supplementary file 1 [file Data_Sheet_1.docx]

Supplementary Material

# Supplementary Tables

# Table 1. Demographic, maltreatment, and vocational background

| Characteristic | | N |
| --- | --- | --- |
| Gender | Male | 7 |
|  | Female | 13 |
| Age at time of interview | 28-34 | 2 |
|  | 35-44 | 8 |
|  | 45-54 | 8 |
|  | 55-60 | 2 |
| Relationship status | Committed relationship | 16 |
|  | Divorced | 2 |
|  | Single | 2 |
| Has children | Yes | 17 |
|  | No | 3 |
| Number of children | 0 | 3 |
|  | 1 | 1 |
|  | 2 | 9 |
|  | 3 | 5 |
|  | 4 | 2 |
| Education level | No higher education | 2 |
|  | Bachelor’s degree | 4 |
|  | Master’s degree or doctorate | 14 |
| Type of employment | Self-employed | 10 |
|  | Salaried employee | 7 |
|  | Both | 3 |
| Managerial positions | No | 10 |
|  | Yes | 10 |
| Type of childhood maltreatment* | Emotional abuse | 15 |
|  | Physical abuse | 10 |
|  | Neglect | 11 |
|  | Sexual abuse | 7 |
|  | Verbal abuse | 8 |
| Socio-economic status in childhood | Low | 8 |
|  | Middle | 10 |
|  | High | 2 |

*More than one type of maltreatment could be reported.**Table 2.** Occupations by participant

| **Pseudonym** | **Current Occupation** |
| --- | --- |
| Adam | Actor |
| Bruce | Social worker (child protection services) |
| Fiona | Therapist |
| Ian | Municipal politician |
| Ilana | Organizational consultant and lecturer |
| Madeline | Gym owner |
| Maya | Lecturer and researcher (humanities) |
| Michelle | Researcher (life sciences) |
| Mickey | Choreographer and dance instructor |
| Mona | Graphic designer |
| Naomi | Social worker |
| Neal | Vice principal, teacher, and therapist |
| Nina | Coach |
| Peter | Eastern medicine healer |
| Shawn | High school principal |
| Sophie | Attorney |
| Tammy | Group facilitator through arts |
| Tara | Teacher |
| Yolanda | Vice principal and teacher |
| Valerie | Artist |

**Interview Protocol**

Part 1

I would like to begin our conversation by talking about your work.

1. Tell me about your current job. What do you do? Where do you work?
2. Tell me about your occupational path and journey up until now. (First job on).
3. Could you tell me about relationships with other people at work?
4. Could you tell me about your relationship with your current supervisor? Can you tell me more about your previous supervisors?
5. How do you see your relationship with your current supervisor in comparison to your relationships with supervisors in the past?
6. Can you tell me more about your occupational choices?
7. For you, what is the meaning of your work?
8. Can you choose a metaphor that describes your current workplace?

Part 2

I would like to go back in time and hear more about your personal history.

1. Could you tell me about the home you grew up in?

2. Could you tell me about your relationship with your father and with your mother?

3. What was it like being a child in your home? What was the experience?

4. Could you tell me what your parents did for a living?

Part 3

1. Where do you see yourself in one year? In ten years?

2. How do you think you will get there?

3. What do you feel inhibits you in your vocational path?

4. What do you feel promotes you in your vocational path?

Part 4: Summary

1. We are interested in the connection between childhood maltreatment and vocational life. We were wondering – do you see any possible links between the two regarding your life?

2. Is there anything you would like to ask me?

Thank you very much!
